# Loss of TRAIL-Receptors Is a Recurrent Feature in Pancreatic Cancer and Determines the Prognosis of Patients with No Nodal Metastasis after Surgery

**DOI:** 10.1371/journal.pone.0056760

**Published:** 2013-02-27

**Authors:** Eike Gallmeier, Dominik C. Bader, Lydia Kriegl, Sabina Berezowska, Hendrik Seeliger, Burkhard Göke, Thomas Kirchner, Christiane Bruns, Enrico N. De Toni

**Affiliations:** 1 Department of Medicine 2, University Hospital Grosshadern, University of Munich, Munich, Germany; 2 Institute of Pathology, University of Munich, Munich, Germany; 3 Department of Surgery, University Hospital Grosshadern, University of Munich, Munich, Germany; University of Torino, Italy

## Abstract

**Introduction:**

Agonistic antibodies targeting TRAIL-receptors 1 and 2 (TRAIL-R1 and TRAIL-R2) are being developed as a novel therapeutic approach in cancer therapy including pancreatic cancer. However, the cellular distribution of these receptors in primary pancreatic cancer samples has not been sufficiently investigated and no study has yet addressed the issue of their prognostic significance in this tumor entity.

**Aims and Methods:**

Applying tissue microarray (TMA) analysis, we performed an immunohistochemical assessment of TRAIL-receptors in surgical samples from 84 consecutive patients affected by pancreatic adenocarcinoma and in 26 additional selected specimens from patients with no lymph nodes metastasis at the time of surgery. The prognostic significance of membrane staining and staining intensity for TRAIL-receptors was evaluated.

**Results:**

The fraction of pancreatic cancer samples with positive membrane staining for TRAIL-R1 and TRAIL-R2 was lower than that of cells from surrounding non-tumor tissues (TRAIL-R1: p<0.001, TRAIL-R2: p = 0.006). In addition, subgroup analyses showed that loss of membrane staining for TRAIL-R2 was associated with poorer prognosis in patients without nodal metastases (multivariate Cox regression analysis, Hazard Ratio: 0.44 [95% confidence interval: 0.22−0.87]; p = 0.019). In contrast, analysis of decoy receptors TRAIL-R3 and -R4 in tumor samples showed an exclusively cytoplasmatic staining pattern and no prognostic relevance.

**Conclusion:**

This is a first report on the prognostic significance of TRAIL-receptors expression in pancreatic cancer showing that TRAIL-R2 might represent a prognostic marker for patients with early stage disease. In addition, our data suggest that loss of membrane-bound TRAIL-receptors could represent a molecular mechanism for therapeutic failure upon administration of TRAIL-receptors-targeting antibodies in pancreatic cancer. This hypothesis should be evaluated in future clinical trials.

## Introduction

Pancreatic cancer is the fourth leading cause of cancer-related death in the industrialized countries with an overall five-year survival averaging less than 5% and a median survival of about six months [1]. Its aggressive biology, the lack of early symptoms and the absence of reliable screening methods are responsible for the advanced stage of this disease at the time of diagnosis. Neither recent improvements in surgical procedures nor radio- or chemotherapeutic regimens have yet led to a significant improvement in patient survival. Consequently, significant effort is being devoted to the development of novel and rational therapeutic approaches that target the critical molecular features in this tumor entity.

In recent years, the administration of agonistic antibodies targeting the receptors for TNF-related apoptosis-inducing ligand (TRAIL) has pre-clinically been demonstrated to represent a promising therapeutic avenue, which is at this time undergoing clinical investigation in numerous tumor entities including pancreatic cancer [2]–[6]. TRAIL plays a role in a wide variety of biological processes including the induction of apoptosis in cancer cells upon binding to its receptors on the outer cell membrane. Of particular importance, it has been shown that apoptosis triggered by TRAIL plays a critical role for tumor surveillance by causing the immune-mediated clearance of metastatic cells; TRAIL-knockout mice exhibit enhanced metastases formation [7], loss of TRAIL receptor-expression is associated with poor prognosis and tumor recurrence in patients suffering from a variety of different tumor types [8], and expression of the TRAIL-binding soluble decoy receptor osteoprotegerin correlates with tumor stage and metastasis formation in colorectal cancer patients [9], establishing a strong rationale for the development of TRAIL-receptors-targeting compounds as therapeutic anticancer strategy.

In spite of the growing interest for TRAIL-receptors as therapeutic target in cancer, studies on the expression, spatial distribution and relevance of TRAIL-receptors as prognostic markers in pancreatic cancer are still lacking. Specifically, little is known about the expression and functional availability of distinct TRAIL-receptors on the surface of pancreatic cancer cells, despite the availability of agonistic antibodies targeting either TRAIL-R1 or TRAIL-R2 currently used in clinical trials. By applying tissue-microarray analyses to evaluate TRAIL-R1 and TRAIL-R2 expression status in a large cohort of surgical specimens of pancreatic adenocarcinoma, we found that loss of membrane staining for these receptors is a common feature of pancreatic cancer. Thus, failure to achieve therapeutic effects using TRAIL-receptors targeting compounds in clinical trials might be due to the lack of selection of the patients with tumors expressing membrane-bound TRAIL-receptors. In addition, loss of membrane-bound TRAIL-R2 in tumors from patients with no nodal metastases at the time of diagnosis is associated with a poor prognosis in our cohort, potentially establishing TRAIL-R2 as a prognostic marker in specific of pancreatic cancer patients.

## Patients and Methods

### Ethics Statement

According to the guidelines of our University, immunohistochemical staining of archived tissue samples may be performed provided that anonymity is granted. Therefore, approval of this study was waived by the ethical committee of the University of Munich and no written consent was required.

### Case Identification, Selection and Patients’ Follow-up

Patients with histologically confirmed pancreatic ductal adenocarcinoma, who underwent surgery for pancreatic cancer (Whipple procedure, distal pancreatectomy, or total pancreatectomy) at the Department of Surgery at the Ludwig-Maximilians-University of Munich between January 31^st^, 2003 and June 14^th^, 2007, were considered for tissue microarray (TMA) construction. As a second step, this patients’ collective was successively broadened by the inclusion of additional 26 consecutive patients with no nodal metastasis undergoing surgery until October 7^th^ 2011 to enable the specific evaluation of the prognostic significance of TRAIL-receptors in determining the risk of recurrence after radical resection. All clinicopathologic data were collected from the database of the Munich Cancer Registry and the original patients’ charts.

In our study, the majority of patients (98 of 110, 89%) received adjuvant therapy including gemcitabine, either as monotherapy (n = 35) or in combination with radiotherapy (n = 50) and/or other agents, including 5-fluorouracil, oxaliplatin and cisplatin.

### TMA Construction

Paraffin-embedded archived tissue material of tumor and surrounding normal pancreatic tissue was used for TMA construction. TMAs were prepared as published before [10]. In brief, the area of interest to be sampled was identified and marked on hematoxylin-eosin stained tissue slides. From the corresponding paraffin block (donor block), tissue core biopsies (each 0.6 mm in diameter) were taken out and then arrayed in a recipient TMA block using a manual arrayer (Beecher Instruments, Sun Prairie, WI). Each case was represented by three core biopsies from different parts of the pancreatic carcinoma and two core biopsies from corresponding normal pancreatic tissue to exclude artefacts due to heterogeneous antigen expression and to allow comparisons between normal exocrine pancreatic tissue and tumor tissue. Immunohistochemistry was performed on 2 µm sections of the TMA.

### Immunohistochemical Staining

5 µm sections of TMA blocks were used for immunohistochemical staining. Anti-TRAIL R1 polyclonal goat antibody (Santa Cruz Biotechnology Inc., Heidelberg, Germany) and Anti-TRAIL R2 polyclonal rabbit antibody (Calbiochem, CA, USA) were applied as primary antibodies. Both antibodies were previously validated for immunohistochemistry of TRAIL-receptors in our laboratory [8]. For antigen retrieval, sections were pre-treated by boiling in a microwave oven 2 times at 15 min at 750 W in Target Retrieval Solution (Dako, Hamburg, Germany). Endogenous peroxidase was blocked by incubation in 7.5% hydrogen peroxide for 10 minutes. Vectastain ABC-Kit Elite Universal (Vector Laboratories, CA, USA) kit was taken for antibody detection and AEC (Zytomed Systems) was used as a chromogen. Slides were counterstained with hematoxylin (Vector). For evaluation of decoy receptors TRAIL-R3 and TRAIL-R4 the following antibodies were used: TRAIL-R3 polyclonal rabbit antibody (Gene Tex, CA, USA) and TRAIL-R4 monoclonal mouse antibody (US Biology, MA, USA). Positive staining for TRAIL-receptors was first categorized according to its cellular distribution and regardless of the intensity of the signal as follows: positive staining in cytoplasm only, positive staining on cell membranes only, and positive staining in both cell membrane and cytoplasm. For statistical analyses, tumors exhibiting TRAIL-receptors staining on cell membranes (“membrane staining” group) were compared to tumors without TRAIL-receptors staining or with TRAIL-receptors expression confined to cytoplasm only (“no membrane staining” group) according to the rationale that TRAIL-receptors are functionally active only if situated on the surface of cell membranes [11]. Additionally, we conducted a semi-quantitative analysis of pancreatic cancer samples by assigning a score depending on the overall TRAIL-receptors staining intensity (0: no staining, 1+ low staining intensity, 2+ high staining intensity).

### Statistics

Overall survival was defined as the interval from the date of surgery to death or to the most recent contact (for censored events) as of September 1^st^, 2012. Categorical data were compared by Chi-Square or Fisher’s exact tests, continuous data were compared by the t-test. Overall median survival times were estimated using the Kaplan-Meier method. The log-rank test was used to test for homogeneity of the survival curves. Univariate and multivariate Cox proportional hazards models were used to assess the effects of variables on overall survival. Follow-up maturity was validated by assessment of follow-up curves for the living patients to ensure comparable follow-up times of survival curves between the respective independent groups [24]. Statistical analyses were performed using Statistical Package for Sciences software (SPSS Inc., Chicago, IL, USA). Two-sided *P* values of less than 0.05 were considered statistically significant.

## Results

### Patient Selection and and Clinicopathological Features

84 consecutive patients with histologically confirmed pancreatic ductal adenocarcinoma, who underwent surgery for pancreatic cancer at the Department of Surgery at the Ludwig-Maximilians-University of Munich between January 31^st^, 2003 and June 14^th^, 2007, were identified. Since the expression of TRAIL-receptors has been shown to be affected by the administration of chemotherapy and radiotherapy [12]–[14], patients who received neoadjuvant chemotherapy or neoadjuvant irradiation were not considered for statistical analyses. Furthermore, patients who died as a result of immediate postoperative complications and patients for whom no follow up was available were excluded.

Where histological data were missing (TRAIL-R1∶5 normal tissue samples; TRAIL-R2∶9 normal tissue samples and 5 tumor tissue samples) only matching samples were considered for the comparison of categorical data between normal and tumor tissues. All patients selected for this study (n = 84) had a complete follow-up either until death (n = 72) or until their most recent contact (n = 12) on September 1st, 2012. The shortest follow-up after surgery for patients still alive as of September 1^st^ 2012 was 62.6 months. The longest follow-up for patients still alive was 106.5 months. The median patient age at the time of surgery was 65 years (range 32–81). A summary of the clinicopathologic features of this patients’ cohort is shown in table 1. As a second step, the patients’ collective was extended by the addition of 26 consecutive patients with no nodal metastasis at the time of surgical intervention who were treated in the subsequent three years’ time period until October 7^th^ 2011. A summary of the clincopathological features of No patients’ subgroup is provided in table S1.

**Table 1:** Summary of clinicopathologic features.

Feature	Patient count	
	n	%
Age, median (y)		
≤65	39	46
>65	45	54
Gender		
Female	43	51
Male	41	49
Tumor differentiation		
Well	1	1
Moderate	27	32
Poor	56	67
Tumor pathologic stage		
T1	2	2
T2	9	11
T3	70	83
T4	3	4
Lymph node status		
No	36	43
N1	48	57
Metastasis status		
M0	73	87
M1	11	13
Tumor size, median (cm)		
<3.5 cm	33	39
≥3.5 cm	51	61
Margin status		
Negative	42	50
Positive	42	50
Postoperative radiotherapy		
No	34	40
Yes	50	60
Postoperative chemotherapy		
No	12	14
Yes	72	86

### Immunohistochemical Staining for TRAIL Receptors 1 and 2 in Pancreatic Cancer and Matched Surrounding Tissues

To assess TRAIL-receptors status in pancreatic cancer samples and matched surrounding tissue, we first conducted a semi-quantitative analysis of TRAIL-receptors-1 and -2 staining intensity regardless of their cellular distribution applying an intensity staining score ranging from 0 to 2.

Positive staining for TRAIL-R1 was found in 77% of tumor samples and 89% of matched surrounding tissue. Instead, positive staining for TRAIL-R2 was found in 99% of both tumor samples and matched surrounding pancreatic tissue (table S2). However, when assessing staining intensity for TRAIL-R2, this receptor appeared to have a lower staining in tumor tissue samples compared to matched non-tumor tissue, with the majority of non-tumor samples showing high intensity-staining (high intensity-staining in non-tumor tissue samples = 71% vs. 48% of tumor tissue samples - McNemar-Bowker test: p = 0.021, table S2). The higher percentage of tumor samples showing a negative staining for TRAIL-R1 and the overall higher intensity staining score for TRAIL-R2 in non-tumor samples indicates a loss of TRAIL-receptors expression in tumor samples vs. matched non tumor tissue samples.

**Figure 1:**
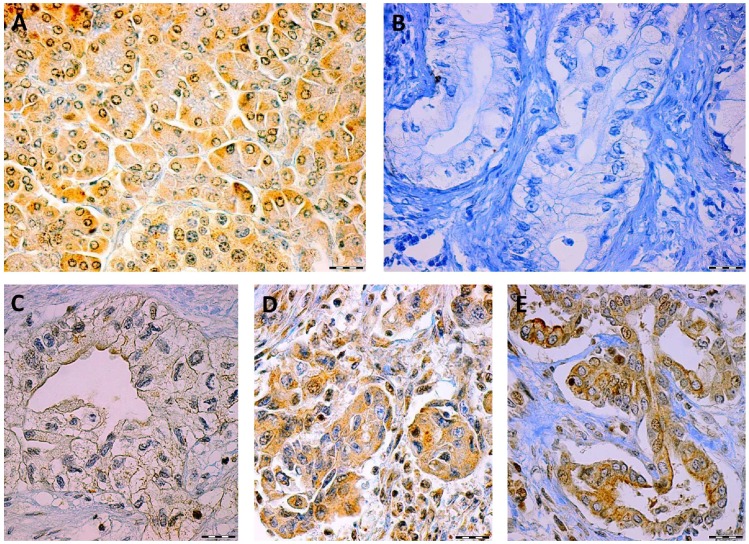
Representative micrographs of TRAIL-R2 staining in normal pancreatic tissue and pancreatic cancer cells showing: typical positive staining of TRAIL-R2 in normal tissue (A), negative staining of TRAIL-R2 in tumor tissue (B) and intracellular spatial distribution of TRAIL-R2 in tumor tissue with prevalent staining of the cell membranes (C), cytoplasm (D) or both (E). Scale bars represent 50 µm.

**Figure 2:**
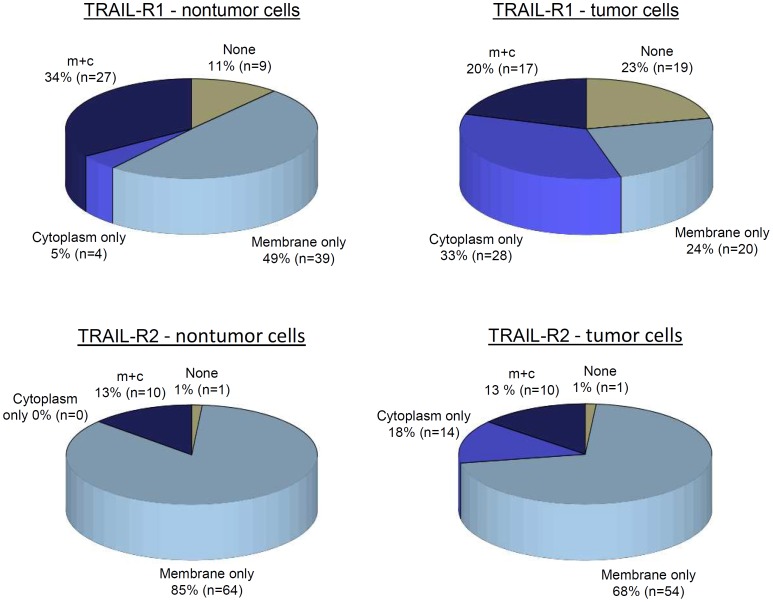
TRAIL-R1 and TRAIL-R2 membrane staining in pancreatic cancer tissue and in surrounding pancreatic cells. Percentage of samples showing no staining (none), cytoplasmatic staining (cytoplasm only), membrane staining (membrane only) or both (m+c).

Next, we analyzed the spatial intracellular distribution of TRAIL receptors by considering the fraction of cells in cancer and surrounding non-tumor tissues according to whether the immunohistochemical analysis showed cytoplasmatic staining, membranous staining or both. Representative figures of different staining patterns of TRAIL-R2 are shown (fig. 1). Importantly, TRAIL-R1 showed positive membrane staining in 84% of normal surrounding pancreatic tissue samples but only in 44% of pancreatic cancer samples, while TRAIL-R2 showed positive membrane staining in 99% of normal and in 81% of pancreatic cancer samples (fig. 2). Thus, the fraction of samples exhibiting membrane staining was significantly lower in cancer than in matched surrounding normal tissues (McNemar test: TRAIL-R1: p<0.001; TRAIL-R2: p = 0.006).

### Correlation of TRAIL-receptors Staining with Clinicopathological Features of Tumor Tissues and Surrounding Normal Pancreatic Tissue

To assess whether TRAIL-receptors staining in tumor samples and matched non-tumor tissue samples was associated with specific clinicopathological features, membrane staining of TRAIL-receptors was correlated to clinical and pathological features of tumors including grading, size and staging of tumors, the presence or absence of tumor-free margins after surgical resection and the post-interventional application of radio- and/or chemotherapy. However, no significant correlations were found between TRAIL-receptors staining on cell membranes in pancreatic cancer (table 2) or surrounding cells (not shown) and any of the above features. Similarly, analyses correlating these parameters with the staining intensity of TRAIL-receptors regardless of their cellular distribution did not show any significant association (data not shown).

**Table 2:** Associations between membrane staining of TRAIL-receptors and clinicopathologic features.

Feature	TRAIL-R1			TRAIL-R2		
	Membrane Staining		*P*	Membrane Staining		*P*
	Negative, n (%)	Positive, n (%)		Negative, n (%)	Positive, n (%)	
Age, median (y)			0.337			0.556
≤65	24 (28.6)	15 (17.8)		6 (7.6)	31 (39.2)	
>65	23 (27.4)	22 (26.2)		9 (11.4)	33 (41.8)	
Gender			0.365			0.110
Female	22 (26.2)	21 (25.0)		5 (6.3)	36 (45.6)	
Male	25 (29.8)	16 (19.0)		10 (12.7)	28 (35.4)	
Tumor differentiation			0.876			0.362
Well and moderate	16 (19.0)	12 (14.3)		3 (3.8)	23 (29.1)	
Poor	31 (36.9)	25 (29.8)		12 (15.2)	41 (51.9)	
Tumor pathologic stage			0.784			0.612
T1 and T2	7 (8.3)	4 (4.8)		2(2.4)	5 (6.3)	
T3 and T4	40 (47.6)	33 (39.3)		13 (16.5)	59 (70.2)	
Tumorsize, median (cm)			0.254			0.386
<3.5	21 (25.0)	12 (14.3)		4 (5.1)	26 (32.9)	
≥3.5	26 (30.9)	25 (29.8)		11 (13.9)	38 (48.1)	
Lymph node status			0.409			0.261
No	22 (26.2)	14 (16.6)		8 (10.1)	24 (30.4)	
N1	25 (29.8)	23 (27.4)		7 (8.9)	40 (50.6)	
Metastasis status			0.102			1,000
M0	38 (45.2)	35 (41.7)		13 (16.5)	55 (69.6)	
M1	9 (10.7)	2 (2.4)		2 (2.5)	9 (11.4)	
Margin status			0.826			0.485
Negative	24 (28.6)	18 (21.4)		6 (7.6)	32 (40.5)	
Positive	23 (27.4)	19 (22.6)		9 (11.4)	32 (40.5)	
Postoperative radiotherapy			0.991			0.603
No	19 (22.6)	15 (17.8)		5 (6.3)	26 (32.9)	
Yes	28 (33.3)	22 (26.2)		10 (12.7)	38 (48.1)	
Postoperative chemotherapy			0.119			0.391
No	4 (4.8)	8 (9.5)		3 (3.8)	7 (8.9)	
Yes	43 (51.2)	29 (34.5)		12 (15.2)	57 (72.1)	

### Prognostic Significance of TRAIL-receptors Staining in Patients Undergoing Surgery

**Figure 3:**
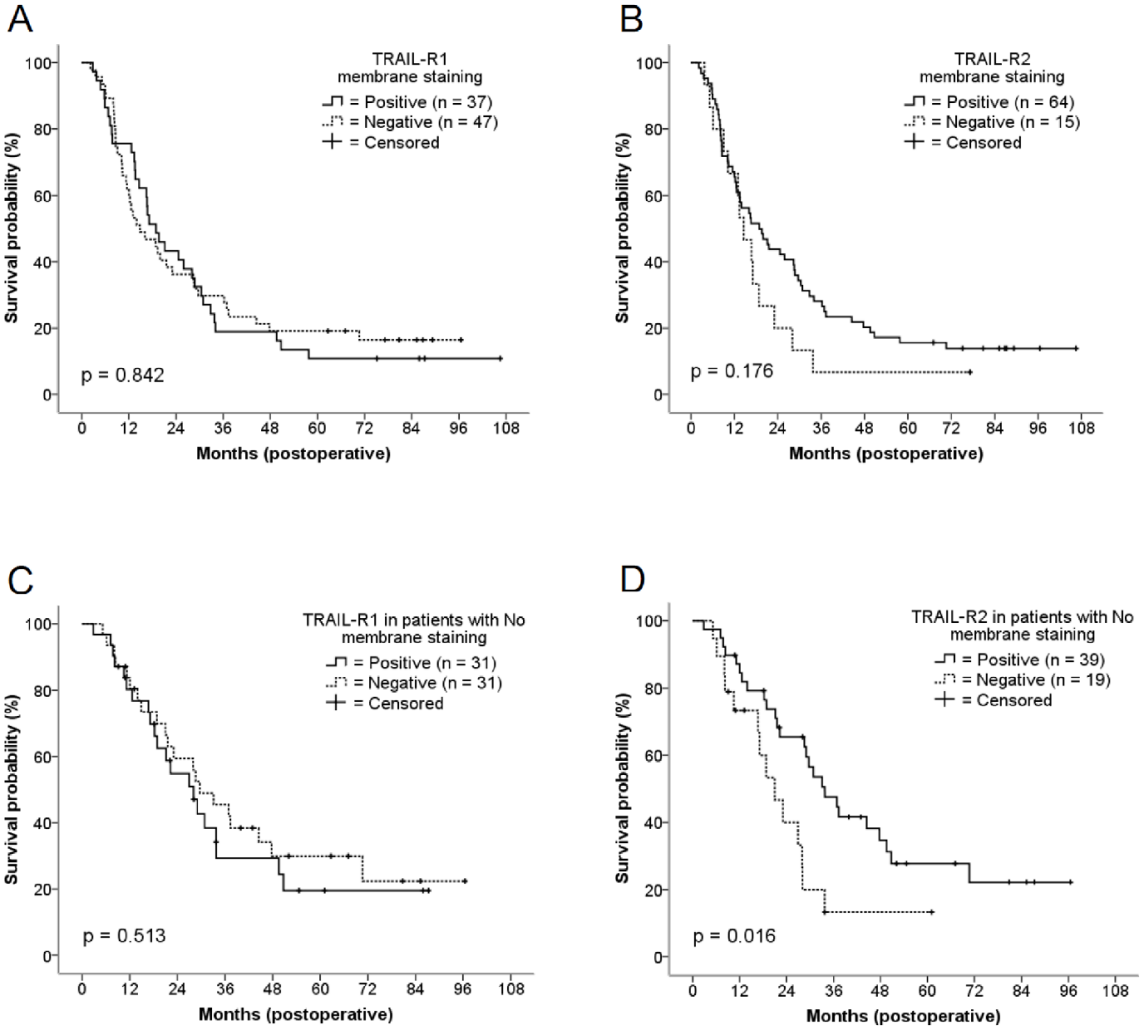
Kaplan-Meier curves of overall survival in patients with resected pancreatic adenocarcinoma. Graphs show survival according to TRAIL-R1 membrane staining (A), TRAIL-R2 membrane staining (B) and subgroup analysis of survival according to TRAIL-R1 (C) and TRAIL-R2 (D) membrane staining in patients with no nodal metastasis at the time of surgery.

As expected, Kaplan-Meier and univariate Cox regression analyses demonstrated lymph nodes (No vs. N1), metastases (M0 vs. M1) and tumor size (median, <3.5 cm vs. ≥3.5 cm) to represent significant determinants of overall survival (figure S1, table 3). In contrast, no correlations were found between overall survival and membranous expression of TRAIL-R1 or TRAIL-R2, respectively (Kaplan-Meier log rank test: TRAIL-R1: p = 0.842, TRAIL-R2: p = 0.176, fig. 3A and 3B). Similarly, analyses stratifying patients according to the semi-quantitative assessment of TRAIL-receptors staining intensity regardless of the cellular distribution (scoring ranging from 0 to 2+) did not yield significant survival differences (Kaplan-Meier long rank test: TRAIL-R1: p = 0.279; TRAIL-R2: p = 0.339– data not shown). However, subgroup analyses revealed that patients without nodal metastasis at the time of surgery had a better prognosis if membrane-bound staining for TRAIL-R2 was positive (HR: 0.30 [0.12–0.76]; p = 0.011– data not shown), whereas no significant associations were found for membrane-bound TRAIL-R1 expression and survival in this subgroup (HR: 1.21 [0.58–2.54], p = 0.608). In support of the data obtained for TRAIL-R2, multivariate analysis including variables associated with overall survival in the univariate analyses with p<0.2, indicated that TRAIL-R2 membrane staining was an independent factor of survival in this subgroup (calculated HR: 0.36 [0.14–0.91], p = 0.031).

**Table 3:** Univariate Analysis of overall survival in patients with resected pancreatic ductal adenocarcinoma.

Variables		No.							Survival*			HR	95% CI										*P*
													Lower					Upper					
Age, median (y)																							
≤65		39							19.4			1.00											0.685
>65		45							16.6			1.06	0.66					1.69					
Gender																							
Female	43								13.1		1.00											0.192	
Male	41								19.6		0.73		0.46					1.16					
Tumor differentiation																							
Well toModerate	28							28.7			1.00											0.259	
Poor	56							14.6			1.32		0.81				2.17						
Tumor size, median (cm)																							
<3.5	33						28.4			1.00											**0.002**		
≥3.5	51						13.1			2.14			1.31				3.50						
Lymph node status																							
No		36					28.1			1.00											**0.015**		
N1		48					13.1			1.80			1.11			2.90							
Metastasis status																							
M0	73					19.6				1.00										**0.002**			
M1	11					10.2				2.83			1.45			5.54							
Margin status																							
Negative		42				18.9				1.00										0.673			
Positive		42				16.1				1.10			0.69		1.75								
TRAIL-R1 membrane staining																							
Negative		47			14.9					1.00									0.842				
Positive		37			18.9					1.05			0.65		1.67								
TRAIL-R2 membrane staining																							
Negative	15				14.6					1.00									0.179				
Positive	64				18.9					0.66			0.36		1.20								
TRAIL-R3 expression																							
Negative	40			14.8						1.00									0.840				
Positive	43			19.3						0.95			0.59	1.51									
TRAIL-R4 expression																							
Negative		26		17.1						1.00									0.806				
Positive		57		16.4						1.06			0.64		1.76								
TRAIL-R1 membrane stainingin patients with No																							
Negative	22			28.6						1.00									0.608				
Positive	14			21.1						1.21			0.58	2.54									
TRAIL-R2 membrane stainingin patients with No																							
Negative		8	17.1							1.00									**0.011**				
Positive		24	30.8							0.30			0.12		0.76								

* Median survival time in months from date of surgery to death or to most recent contact.

To substantiate our results on the No population, we subsequently extended the size of this subgroup to include 26 additional No patients who underwent surgery for pancreatic cancer in the three years period following that of the previous TMA collective. Analysis of survival in this extended subset of altogether 58 patients confirmed that only membrane staining of TRAIL-R2 was associated with a better survival (univariate analysis: TRAIL-R1: HR 1.22 [0.66–2.25], p = 0.514; TRAIL-R2 (HR 0.45 [0.22–0.88], p = 0.019– figure 3 C, D). Multivariate analysis, including variables associated with overall survival in the univariate analyses with p<0.2, (i.e. gender, grading, median tumor size, and TRAIL-R2 membrane staining) confirmed the independent prognostic significance of membrane staining for TRAIL-R2 in this subgroup (adjusted for all: HR 0.47 [0.23–0.96], p = 0.041; backward elimination: HR 0.44 [0.22−0.87], p = 0.019).

### Staining and Cellular Distribution of Decoy Receptors TRAIL-R3 and TRAIL-R4 in Pancreatic Cancer Cells

Decoy receptors for TRAIL have shown to play a role in the pathogenesis of tumors as revealed by reports published previously by our and other laboratories [9], [15], [16], but presently no information on the prognostic significance of decoy receptors for TRAIL in pancreatic cancer is available. To investigate the potential role of these receptors in antagonizing the effects of TRAIL-R1 and -R2, additional analyses were performed and TRAIL-R3 and TRAIL-R4 staining in tumor samples of our patients’ collective assessed. In agreement with the previous reports, our analyses showed positive TRAIL-R3 and TRAIL-R4 staining in 52% and 69% of tumor samples respectively, and a high-intensity staining in 10% and 16% of cases respectively. However, the staining of these receptors was restricted exclusively to the cytoplasm and had no prognostic significance in our cohort (TRAIL-R3: HR 0.95 [0.59–1.51], p = 0.840; TRAIL-R4: HR 1.06 [0.64–1.76], p = 0.806).

## Discussion

### Targeted Therapies and TRAIL-signaling in Pancreatic Cancer

The recognition of the role played by TRAIL-mediated apoptosis in the process of immune surveillance counteracting tumor formation has led to the development and clinical employment of TRAIL-receptors targeting compounds as anticancer therapy; at this time, monoclonal antibodies targeting TRAIL-R1 (e.g. mapatumumab [17]) or TRAIL-R2 (e.g. tigatuzumab [18]), or recombinant forms of human TRAIL [19], are undergoing extensive clinical investigation. Although the mere expression of receptors for TRAIL does not represent the only determinant of response to the apoptotic effect of TRAIL [20], it is likely that the efficacy of these compounds will act in a TRAIL-receptor expression-dependent fashion in individual tumors. Surprisingly however, while the frequent loss of TRAIL-receptors reported for several tumor entities could impede the clinical efficacy of these compounds [8], [21]–[23], no large systematic report on the spatial cellular distribution and prognostic meaning of TRAIL-receptors in pancreatic cancer is yet available (reviewed in [3]). Furthermore, while early phase clinical trials with TRAIL-receptors-targeting compounds or antibodies have just recently been initiated in patients with pancreatic cancer, the results from clinical trials with TRAIL-receptors-targeting compounds in colorectal and non-small-cell lung cancer have shown disappointing results [17], [19], prompting a timely clarification of the role of these agents in the therapy of solid tumors.

### Staining and Intracellular Localization of TRAIL-receptors in Tumors Versus Surrounding Pancreatic Tissues

Considering the overall staining of tumor and matched normal pancreatic samples, we found that almost all samples stained positive for TRAIL-R2; instead, TRAIL-R1 stained negative in 23% of tumor samples and in 11% of matched normal pancreatic samples. The loss of TRAIL-R1 staining in tumor cells in comparison to co-stained surrounding non-tumor tissues from the same patient is in agreement with previous reports on other tumor entities [23], [24] and with *in vitro* evidence showing that loss of TRAIL-receptors in pancreatic cancer cell lines contributes to a decreased sensitivity towards TRAIL-induced apoptosis [25]. However, the only other published study on the prevalence of TRAIL-receptors in pancreatic cancer demonstrated upregulation of TRAIL-R1 in 38 pancreatic cancer samples as compared to 31 non-malignant pancreatic ductal tissue samples from control patients [26]. Several explanations exist for the different results obtained in this study versus our study, including a lower patient number in the other cohort, the source and representativeness of the samples (partly biopsies in the other study versus solely surgical specimens in our study), and the inclusion (11 of 34 patients) versus exclusion of patients having received neo-adjuvant therapy, together with the fact that the expression of TRAIL-receptors is increased by chemo−/or radiotherapy [27], [28].

Most previous reports on TRAIL-receptors expression in different tumor entities were based on semi-quantitative criteria [22], [26], [29]; however, recent studies have shown that particularly the sub-cellular localisation of TRAIL-receptors might influence their function; specifically, while internalization of the pro-apoptotic receptor CD95 plays a stimulatory role in the transduction of apoptotic signalling, internalization of TRAIL-receptors inhibits caspase activation [30]. We recently proposed that TRAIL-receptors expression should be evaluated by analyzing the fraction of membrane-bound TRAIL-receptors, based on the rationale of assessing the fraction of TRAIL-receptors effectively exposed to circulating TRAIL as supposedly predominant prognostic determinant [8]. Accordingly, we considered not only the staining intensity, but also the intracellular distribution of staining, i.e. the fraction of tumors exhibiting TRAIL-receptors staining on cell membranes in our study. We found that 56% of tumor samples displayed no membrane staining for TRAIL-R1 and 19% for TRAIL-R2 with the extent of membrane staining varying inversely with the cytoplasmatic staining, suggesting that internalization of TRAIL-receptors could represent a mechanism for the loss of functional TRAIL receptors as a distinctive feature of pancreatic cancer cells, a hypothesis recently corroborated by other studies *in vitro* [11], [31].

### Prognostic Impact of TRAIL-receptors Expression in Pancreatic Cancer

To assess whether loss of membrane-bound TRAIL-receptors might have prognostic significance, we subsequently correlated TRAIL-receptors staining with survival. Five-year survival of patients harboring tumors exhibiting TRAIL-R1 or TRAIL-R2 did not significantly differ from that of other patients. Similar results were obtained by stratifying patients according to TRAIL-receptors intensity staining scores. However, membrane staining for TRAIL-R2 was associated with a better prognosis in a subgroup of patients without nodal metastases at the time of surgery, which is consistent with recent evidence that the fraction of membrane-bound receptors determines their functional status [11], [31] and plays a major prognostic role in patients with hepatocellular carcinoma [8].

Although we found no specific correlation between TRAIL-receptors expression and tumor stage in our cohort, the fact that TRAIL-receptors expression was not associated with any pathological parameter supports the hypothesis that, rather than influencing tumor initiation, loss of TRAIL-receptors might affect tumor progression at a later stage due to the selection of cell clones resistant to circulating TRAIL and to immune-mediated mechanisms controlling clearance of metastatic cells [32], [33]. Thus, the lack of correlation between TRAIL-receptors status and prognosis in patients with metastatic disease at the time of surgery is likely ascribable to other predominating metastasis-determining factors outweighing the effects of TRAIL-receptors loss at later stages. Recent reports have shown that apoptosis is preferentially triggered in pancreatic cancer cells by TRAIL-R1 especially in combination with XIAP inhibitors, whereas stimulation of TRAIL-R2 requires cross-linking for efficient induction of apoptosis [34]. These data suggest that TRAIL-R1 should be the preferential target of TRAIL-R-targeting agents in pancreatic cancer. Our data showing a lower prevalence of membrane staining of TRAIL-R1 vs. TRAIL-R2 seem to confirm the fact that loss of functional TRAIL-R1 represents an important step of carcinogenesis. However, the prognostic relevance of TRAIL-R2 in patients with no metastasis at the time of surgery suggests that this receptor might exert a physiological role as a tumor-suppressor at this stage of tumor development.

In opposition to the positive prognostic significance of TRAIL-R2, we found no correlation between TRAIL-R3 or TRAIL-R4 and survival. Decoy receptors for TRAIL have shown to play a role in the pathogenesis of tumors as revealed by reports on the detrimental prognostic effect of TRAIL-R3 expression in colorectal cancer [15] or of TRAIL-R4 in breast cancer [16], and by our previously published data on the role of the soluble decoy receptor OPG in determining the resistance to apoptosis in colorectal cancer patients [9]. Our finding that TRAIL-R3 and TRAIL-R4 staining in tumor samples is found exclusively in the cytoplasm is in agreement with the previous finding that positive staining for TRAIL-R1 and -R2, but negligible staining for TRAIL-R3 and -R4 could be found on the surface of different pancreatic cancer cell lines by FACS analysis [34]. Future studies will have to assess whether the absence of membrane staining for TRAIL-R3 and -4 might reflect the lack of prognostic significance of these receptors in pancreatic cancer in contrast to the tumor entities where a negative correlation with survival was shown.

### Clinical Consequences of TRAIL-receptors Loss in Pancreatic Cancer Cells

A clinical consequence of the frequent functional loss of TRAIL-receptors in pancreatic cancer may be represented by the fact that many pancreatic tumors do not respond to the administration of the specific agonistic antibodies targeting either TRAIL-R1 such as Mapatumumab or TRAIL-R2 such as Tigatuzumab [27]. Therefore, clinical trials based on the administration of such agents might have to take the status of the respective membrane staining into account.

The mechanisms by which TRAIL-receptors are lost in tumor cells are not yet fully understood. Genetic loss or mutation of TRAIL-receptors is a rare event in cancer cells, averaging 1% in hepatocellular carcinoma [21], [35], and being absent in pancreatic cancer in large-scale comprehensive genetic analyses [36], [37]. Furthermore, as several compounds [11], [38]–[42] can increase TRAIL-receptors expression or exposure on cell membranes, epigenetic gene silencing and TRAIL-receptors internalization indicate a potentially reversible mechanism for the loss of functional TRAIL-receptors in cancer. Importantly, it has been recently shown that silencing HuR augments TRAIL-R2 production by enabling its translation, hereby enhancing TRAIL-mediated apoptosis [43]. Thus, tumors displaying negative membrane staining for TRAIL-receptors might be susceptible to agents capable of increasing TRAIL-receptors expression, thereby restoring the efficacy of endogenous TRAIL [44], [45]. Also, TRAIL-receptors agonistic antibodies might synergize to induce apoptosis in combination with agents capable of increasing TRAIL-receptors expression or overcome intracellular resistance to TRAIL [3], [12], [43], [45], [46].

In conclusion, this pilot study shows that loss of membrane-bound TRAIL-receptors could represent a molecular mechanism for therapeutic failure upon administration of TRAIL-receptors-targeting antibodies in pancreatic cancer. TRAIL-R2 might represent a prognostic marker for patients with early stage disease. Immunohistochemical analysis of prospectively collected tissues in the context of current clinical trials using TRAIL-receptors agonistic antibodies will provide definitive evidence on the importance of these receptors and of their spatial distribution in determining the prognosis of pancreatic cancer patients, and on their relevance as a biomarker of response to these novel anti-cancer compounds.

## Acknowledgments

The authors are thankful to Dr. Alexnder Crispin and Mr. Rüdiger Laubender for precious discussion of statistical analysis.

## Supporting Information

Figure S1 **Kaplan-Meier curves of overall survival in patients with resected pancreatic adenocarcinoma.** Graphs show survival according to the median tumor size (A), lymph node status (B) and metastasis status (C).
(TIF)

Figure S2 **Representative micrographs of TRAIL-R3 staining in pancreatic cancer cells showing: (A) no staining of TRAIL-R3 with scattered positive lymphocytes (magnification ×630), (B) weak staining intensity of TRAIL-R3 (magnification ×400) and (C) strong staining intensity of TRAIL-R3 (magnification ×630).**
(TIF)

Table S1 **Summary of clinicopathological features of patients with no nodal metastasis at the time of surgery.**
(XLS)

Table S2 **Staining intensity of TRAIL-R1 and TRAIL-R2 in tumor- and matched non-tumor tissue samples.**
(XLS)
