# Supplementary figures and images for: Loss of TRAIL-Receptors Is a Recurrent Feature in Pancreatic Cancer and Determines the Prognosis of Patients with No Nodal Metastasis after Surgery
Source: PLoS One. 2013 Feb 27;8(2):e56760. doi: 10.1371/journal.pone.0056760 (PMC3584093; doi:10.1371/journal.pone.0056760)

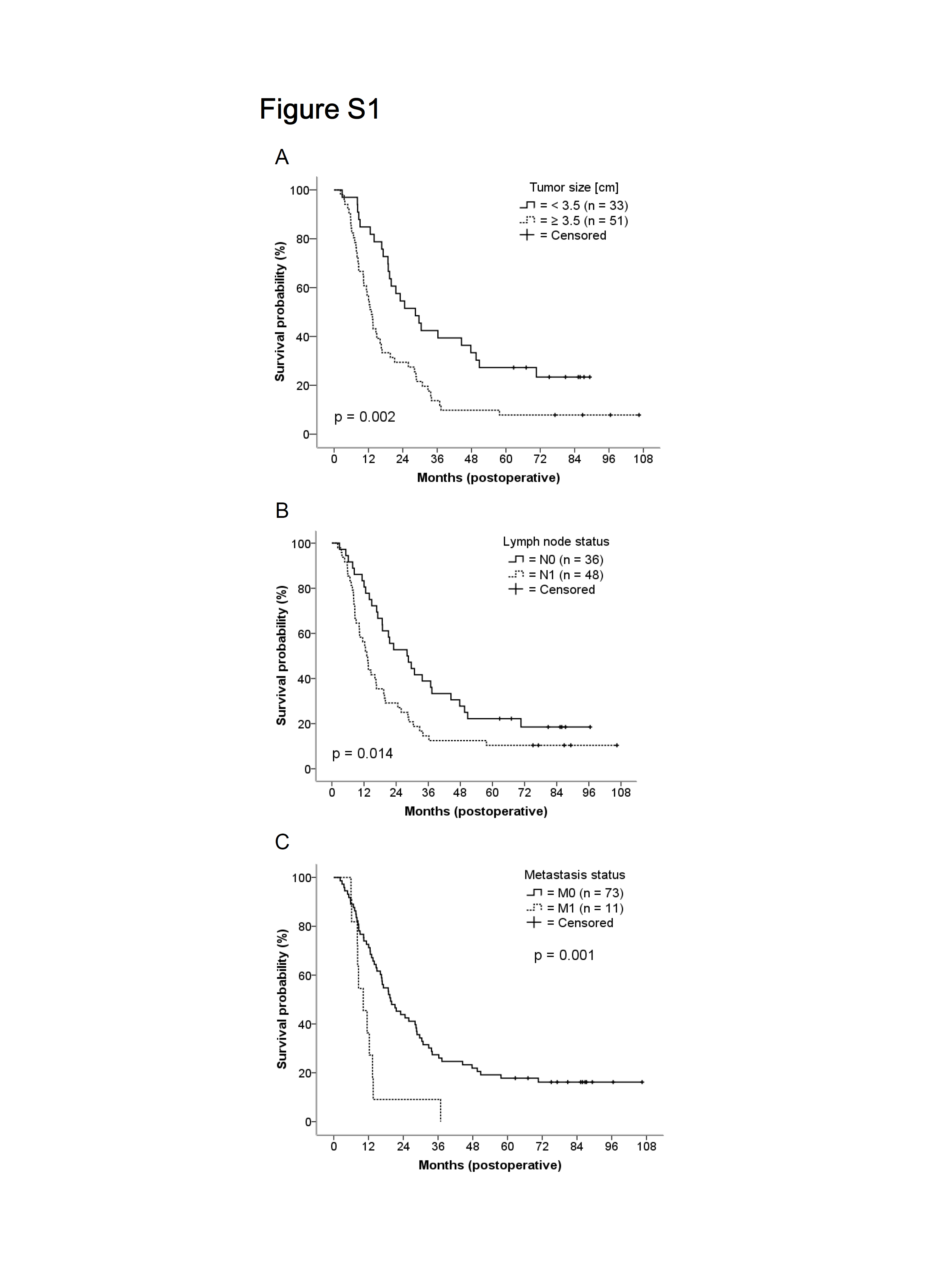

Supplement: Figure S1 — Kaplan-Meier curves of overall survival in patients with resected pancreatic adenocarcinoma. Graphs show survival according to the median tumor size (A), lymph node status (B) and metastasis status (C). (TIF) [file pone.0056760.s001.tif]

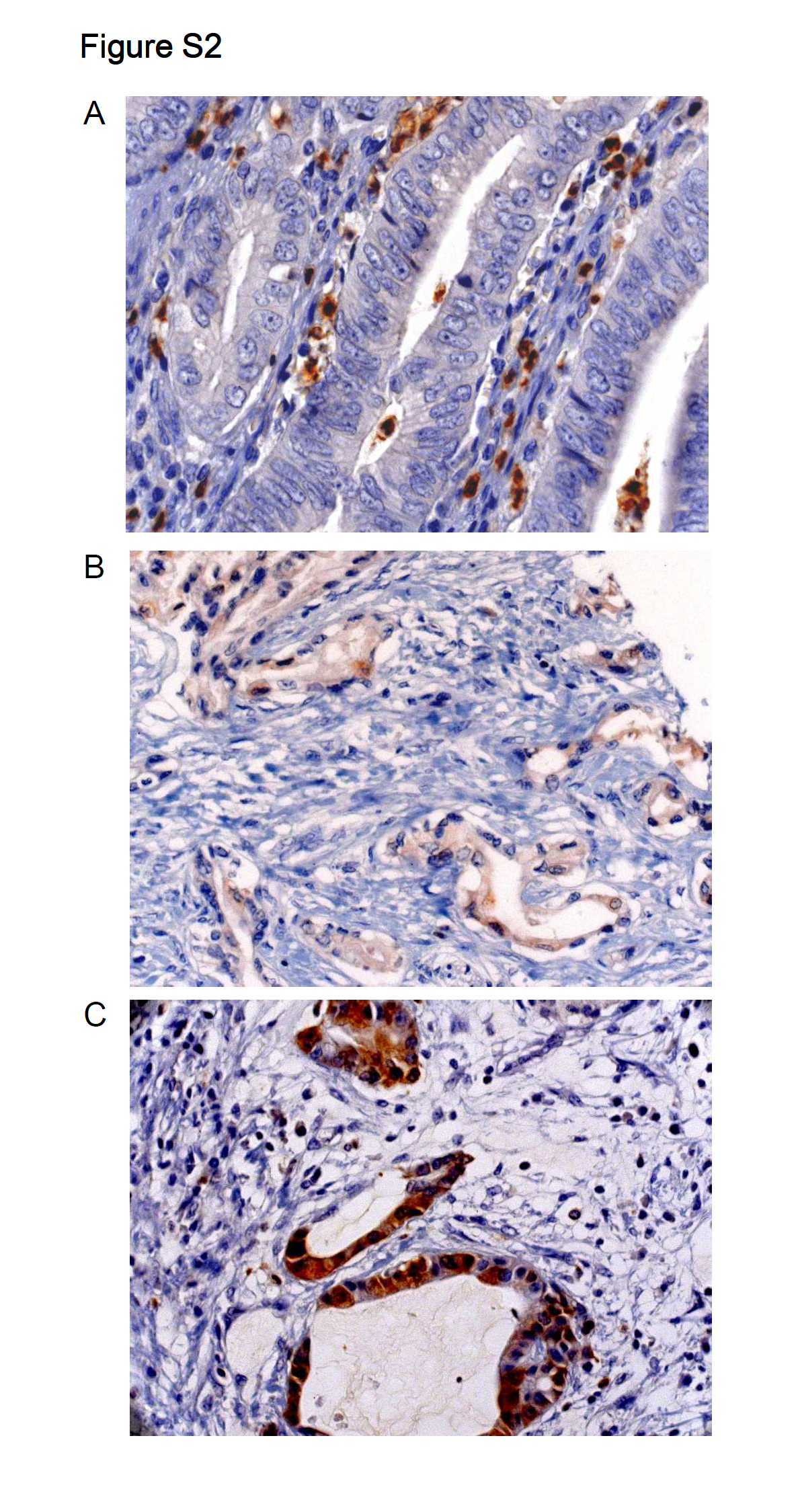

Supplement: Figure S2 — Representative micrographs of TRAIL-R3 staining in pancreatic cancer cells showing: (A) no staining of TRAIL-R3 with scattered positive lymphocytes (magnification ×630), (B) weak staining intensity of TRAIL-R3 (magnification ×400) and (C) strong staining intensity of TRAIL-R3 (magnification ×630). (TIF) [file pone.0056760.s002.tif]
